# Light-Emitting Diodes Modify Medicinal Quality of Mown *Rabdosia rubescens*, with Changes in Growth, Physiology, and Antioxidant Activity, under Drought Stress

**DOI:** 10.3390/plants12183189

**Published:** 2023-09-06

**Authors:** Jun Gao, Ping Meng, Yan Zhao, Jinsong Zhang, Chunxia He, Qirui Wang, Jinfeng Cai

**Affiliations:** 1 Key Laboratory of Tree Breeding and Cultivation, National Forestry and Grassland Administration, Research Institute of Forestry, Chinese Academy of Forestry, Beijing 100091, China; gaojun@caf.ac.cn (J.G.); mengping@caf.ac.cn (P.M.); zhangjs@caf.ac.cn (J.Z.); 2 Collaborative Innovation Center of Sustainable Forestry in Southern China, Nanjing Forest University, Nanjing 210037, China; caijinfeng1984@126.com; 3 Henan Xiaolangdi Forest Ecosystem National Observation and Research Station, Jiyuan 454650, China; 4 College of Horticulture and Plant Protection, Henan University of Science and Technology, Luoyang 471023, China; zhaoyanvip2008@163.com; 5 School of Landscape Architecture and Art, Henan Agricultural University, Zhengzhou 450002, China

**Keywords:** *Rabdosia rubescens*, LED, artificial illumination, water deficit, medicinal quality

## Abstract

Medicinal plants accommodated by understory habitats can easily suffer over-exploitation in the heavy harvest of natural products. It is necessary to develop a sustainable cultural protocol to provide high-quality stocks for efficient regeneration. Drought places stress on medicinal plants during their culture by limiting new sprout growth and reducing the quality of medicinal extracts. Artificial mediating approaches should be considered in a sustainable regime of medicinal plant culture to test the potential tradeoff between resistance to drought and production ability. In this study, *Rabdosia rubescens* seedlings were raised in three light-emitting diode (LED) spectra from red (71.7% red, 14.6% green, 13.7% blue), green (26.2% red, 17.4% green, 56.4% blue), and blue (17.8% red, 33.7% green, 48.5% blue) lights. Mown seedlings were subjected to a simulated drought event. Drought stressed the seedlings by reducing the growth, dry mass, nitrogen (N) uptake, and oridonin content. Mowing increased the oridonin content but decreased total C and N accumulation and the δ^13^C level. The red light benefitted starch accumulation only under the well-watered condition, and the green light induced an upregulation of δ^13^C but decreased antioxidant activity. Oridonin content was negatively associated with combined δ^13^C and catalase activity. Overall, either mowing or blue light can be recommended for the culture of *R. rubescens* to increase oridonin content, alleviating some of the negative consequences of drought.

## 1. Introduction

Global climate change is increasing the frequency of droughts, which further modifies air CO_2_-O_3_ concentrations, reduces precipitation, increases risks of water loss through transpiration, leads to the accumulation of high-temperature records, and promotes more extreme weather events [1,2]. Drought reduces dry mass production and limits physiological activities in medicinal plants, which, together, result in production loss and quality degradation [1,3,4]. In recent years, the frequency of drought-associated ecological risks has increased up to a level that may have led to 7% or higher production damage for global crop production and 8–11% or even more in developing countries [5]. More knowledge is needed about the effects of drought on plant physiology to cope with the increasing risks of water deficiency in a changing world.

Wild plant resources can be introduced to artificially constructed habitats and tamed to acclimatize to water deficit shocks [6,7]. Medicinal plants usually suffer severe exploitation because they are harvested for secondary metabolites that have high medicinal value, placing a heavy burden on the reserves of natural populations. Hence, their juvenile stocks are usually cultured in the nursery to shape high-quality stocks for transplant to quickly regenerate a destroyed population at the understory layer. As abiotic stress, drought threatens plant growth and development by impairing botanic functions in the secondary metabolism [8,9,10], photosynthetic physiology [9,10], nutrition [2,8], and antioxidation [8]. Plants can perceive these changes and adjust themselves in multiple ways, resulting in essential adaptations to drought [11,12]. For medicinal plants, the process of drought adaptation is associated with changes in not only medicinal parameters but also eco-physiological variables. These plants are cultured in this way for several reasons; not only is it expected to improve the quality of medicines [8,9,13], but it can also promote drought resistance [4,14]. Therefore, the relationship between these two types of changes has a practical meaning for predicting changes in medicinal parameters by monitoring other plant responses. 

Drought exerts a strong effect on growth and development in the shoot sprouting of annual plants [14]. Sprouted new shoots can be harvested as a pool of accumulative secondary metabolites [15,16,17,18]; hence, drought may reduce the amount of harvested medicinal materials by reducing the total dry mass production in annual shoots. However, perennial roots reserve essential substances that can support and fuel annual shoot sprouting. Mowing has been proven to be a feasible practice that can promote annual sprouting from perennial roots help the plants to cope with the shock of drought’s effects [19,20]. Mowing forage plants can improve their water-use efficiency through modulating stomatal conductance and gas exchange [19]. It has been identified that mowing can increase the synthesis and accumulation of secondary metabolites in annual shoots of medicinal plants [16]. As a simple manipulation treatment, mowing can also be incorporated into the cultural protocol for medicinal plants in scenarios posing a higher probability of drought events. 

Illumination is a strong driver of plant growth and development. Understory sunlight spectra modify leaf nutrition and carbohydrate contents and determine medicinal plant quality [16,21]. People modify the spectra of lighting for medicinal plants by supplementing with artificial illumination. Accumulating evidence has revealed that the light spectrum has a strong effect on key parameters of plant quality [22,23]. The lighting spectrum can modify the water content and reserves of substances that confer drought resistance [17]; hence, a change in light spectra can mean a switch in plant tolerance to drought [24,25]. LED light can be flexibly adjusted to create and change lighting spectra as needed for medicinal plant culture. For example, a spectrum with a 70%:30% red-to-blue light ratio can increase resistance to drought stress in lemon balm (*Melissa officinalis*) plantlets [24]. LED lighting was found to interact with mowing, and both factors had combined effects on the development of newly sprouted shoots [16,19] and nutrient utilization [16]. The best strategy for setting up a solid spectrum of artificial light with other cultural practices to help plants cope with drought is still undetermined. It is necessary to clarify the relationship between the quality of medicinal plants and eco-physiological parameters and take this into account when designing artificial lighting regimes when facing a scenario of global climate change. 

When drought has negative effects on C_3_ plants, it is perceived as a signal that depresses leaf stomatal conductance [19]. Gas exchange is reduced accordingly with the downregulation of transpiration, and intercellular CO_2_ accumulates to a higher concentration than that experienced with ordinary gas exchange [4]. This increases the ratio of the heavier carbon (C) isotope (^13^C) against the lighter one (^12^C), forming a C isotope signature (*δ*^13^C) in newly formed photosynthates [4,19]. Therefore, more positive δ^13^C is usually found following severe limits on stomatal conductance and a decline in gas exchange, which could indicate changes to water-use efficiency and acclimation to drought [26,27,28]. This theory of plant physiology has been verified in woody plants, such as conifer trees [4,28], fruiting shrubs [27], and several genotypes of alfalfa [19,26]. Therefore, the parameter of *δ*^13^C has the full potential to be used as a flexible predictor of acclimation to drought in medicinal plants subjected to multiple manipulations.

*Rabdosia rubescens* (Hemsley) H. Hara “contains many active ingredients such as terpenoids, flavonoids, polysaccharides and organic acids”, which, together, result in the effects of “heat-clearing and detoxicating, antibacterial and anticancer, promoting blood circulation to arrest pain and anti-tumor” [29]. Annually, sprouted shoots are the main organ in *R. rubescens* that contain natural compounds with desired amounts for medicinal uses [30]. Oridonin is a key parameter that can be extracted from *R. rubescens* and used to inhibit tumor growth [31], delay the aging process [32], and counter cancer cell enlargement [33]. Hence, the oridonin content has been used to assess the medicinal quality of *R. rubescens* [34,35]. In this study, *R. rubescens* seedlings were cultured to test the combined effects of mowing, drought, and LED spectra on eco-physiological parameters in this plant species. The objective of this study was to develop a cultural regime that could alleviate the impairment of oridonin production caused by drought stress in *R. rubescens*. The other objective of this study was to put forth a model predicting oridonin content by regressing against the combined plant variables of growth, dry mass, physiology, δ^13^C, antioxidant activity, and gas exchange. 

## 2. Results and Discussion

### 2.1. Weight of Potted Seedlings Exposed to Drought

*Rabdosia rubescens* seedlings showed a greater weight in red light under a well-watered condition (Figure S1). Seedlings exposed to blue light under a controlled condition also showed high pot weight, although the weight of the plants subjected to this treatment was lower than that for controlled red-light-exposed seedlings at 39 and 53 days of the experiment. Drought-treated seedlings generally showed lower pot weights, although not always with a significant difference, which increased from 63 days to 77 days after the transplant (Figure S1). 

The change in pot weight between two consecutive days indicates a fluctuant dynamic (Figure S1). In the period of 39–53 days of the experiment, the change in pot weight declined under well-watered conditions when the plants were exposed to red and blue lights. Thereafter, the change in pot weight immediately increased to a level between 100 and 200 g between days 53 and 63. The second increase occurred on days 98–105 of the experiment, when drought-exposed seedlings showed high increments in pot weight up to a level between 50 and 150 g. 

Results of this study suggest that drought stress impaired the accumulation of dry mass; hence, the pot weight of stressed seedlings was generally lower than that of well-watered ones. The difference in pot weight between the well-watered control and drought stress treatment plants increased significantly with time because the pot weight in stressed seedlings tended to decline, especially during the later stages of the experiment, with the green light. These findings agree with those reported in sessile oak (*Quercus petraea*) seedlings [36], and both can be attributed to the accumulative effects of hydraulic conductance impairment at a level that suspends growth. Seedlings were mown 46 days after transplant when the pot weight of the well-watered seedlings exposed to red and blue lights suddenly decreased sharply. The sudden declines in the pot weight of seedlings subjected to red and blue lights at 39–53 days could be accounted for by two possible explanations. One may put forth that the plants exposed to red and blue lights controlled their gas exchange at this growing stage: a cost at which other inner substances were being shaped and accumulated [37]. The other possibility is that red and blue lights stimulated photosynthetic performance, and transpiration was activated to a larger extent than was the case with other light spectra [38]. More evidence is needed to monitor photosynthetic and transpiration and identify the validity of either possible explanation. 

### 2.2. Growth, Oridonin Content, and Dry Mass

**Figure 1:**
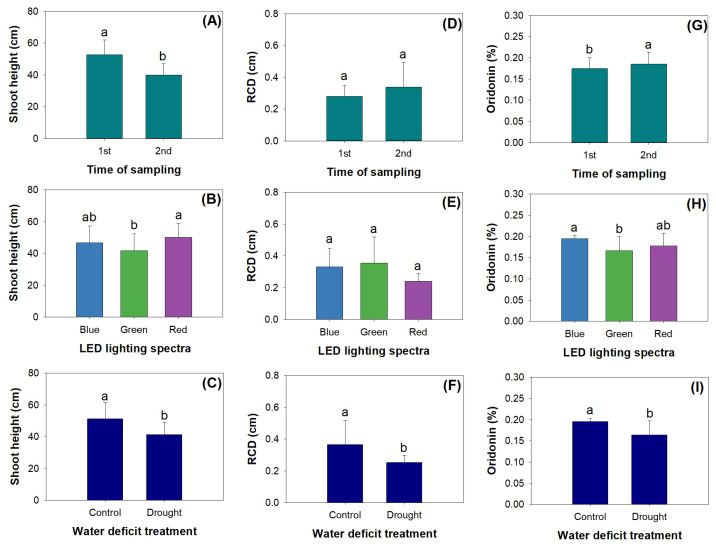
Shoot height (**A**–**C**), root–collar diameter (RCD) (**D**–**F**), and shoot oridonin content (**G**–**I**) in *Rabdosia rubescens* seedlings exposed to combined mowing (1st vs. 2nd samplings), LED spectra (blue, green, red lights), and water deficit treatment (well-water control vs. drought). Different letters marked above error bars (standard errors) represent significant differences according to Tukey’s test at the 0.05 level.

Mowing, LED spectra, and drought factors had main effects on seedling height and oridonin content in shoots, but only drought modified the root–collar diameter (RCD) (Table 1). Mown plants had a lower shoot height compared to unmown plants (Figure 1A). By contrast, mowing increased the oridonin content in shoots (Figure 1G). The red light resulted in a higher shoot height than the green light, but the blue light did not modify the shoot height differently from the other two lights (Figure 1B). The blue light resulted in higher oridonin content than the green light; the contents under both types of light were not significantly different from the content under the red light (Figure 1H). Drought reduced the shoot height, RCD, and oridonin content relative to the well-watered control (Figure 1C,F). 

**Figure 2:**
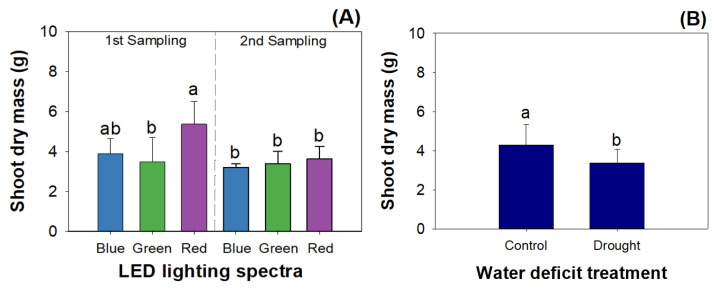
Combined treatments of LED spectra and mowing (**A**) and the unique effect of water deficit treatment (**B**) on shoot dry mass weight in *R. rubescens* seedlings. Different letters marked above error bars (standard errors) represent significant differences according to Tukey’s test at the 0.05 level.

Mowing and LED spectra had an interactive effect on shoot dry mass, while drought exerted the main effect on it (Table 1). The red light resulted in a greater shoot dry mass than the green light before mowing. The shoot dry mass in red-light-exposed seedlings before mowing was also greater than that in mown seedlings subjected to all three types of LED lights (Figure 2A). Drought reduced the shoot dry mass more than the control (Figure 2B).

In this study, *R. rubescens* seedlings were sensitive to water deficit stress, which manifested as declines in their shoot growth, dry mass, and oridonin content under drought. A decline in fat accumulation was also reported in alfalfa [19]. Mowing limited shoot elongation but increased medicinal quality by stimulating the accumulation of oridonin. The decline in shoot growth caused by mowing resulted in an alternative increase in the oridonin content because dry mass production was limited while fat synthesis was not affected. Mowing was also reported to reduce plant community height in the typical steppe grasslands of Inner Mongolia [39]. However, mowing was also found to promote shoot growth in *Aralia elata* seedlings [16]. In addition, mowing had an inverse effect on alfalfa (*Medicago sativa*), which reduced the oridonin content in mown shoots [19]. The difference in effects among these studies resulted from the variation in mowing frequency and intensity [39]. Mowing did not generate any combined effects with drought on shoot growth and oridonin content, which suggests that mowing cannot modify the plasticity of *R. rubescens* to acclimate in drought. 

In this study, oridonin content was determined from *R. rubescens* leaves under a controlled environment where illumination was provided by LED lighting. Oridonin content ranged from 0.10 to 0.21%, with an average of 0.18 ± 0.02% (percent ± standard error). This level is much lower than that collected from *R. rubescens* in fields (averaging 0.544 ± 0.061%) (Table 2). However, oridonin content was comparable with that determined from the leaves of *Isodon enaderianus* under field conditions (averaging 0.172 ± 0.185%), especially in Hubei (0.160%) and Henan (0.174%) (Table 2). Thus, oridonin content in this study was lower than the expectation that originates from records obtained from the same species under field conditions. The field condition is much harsher for establishing and growing *R. rubescens* due to varied kinds of stressors. The synthesis of oridonin, a secondary metabolite, is upregulated under harsh conditions in this environment [40]. In this study, indoor condition was much milder and lacked a strong stressor that would stimulate oridonin synthesis. However, oridonin content in this study fell within a reasonable range for *Isodon enaderianus* plants, even in comparison with their records collected under the field condition. Overall, oridonin content could be accepted as the outcome for *R. rubescens* obtained from a controlled environment and subjected to LED lighting. After all, results herein were obtained from a scientific experiment exploring the response to LED lighting combined with drought and mowing. results in this study can be referred to for future work if LED lighting is proposed for use in field cultivation. 

### 2.3. Physiology, Carbohydrate, and Nitrogen Responses

**Figure 3:**
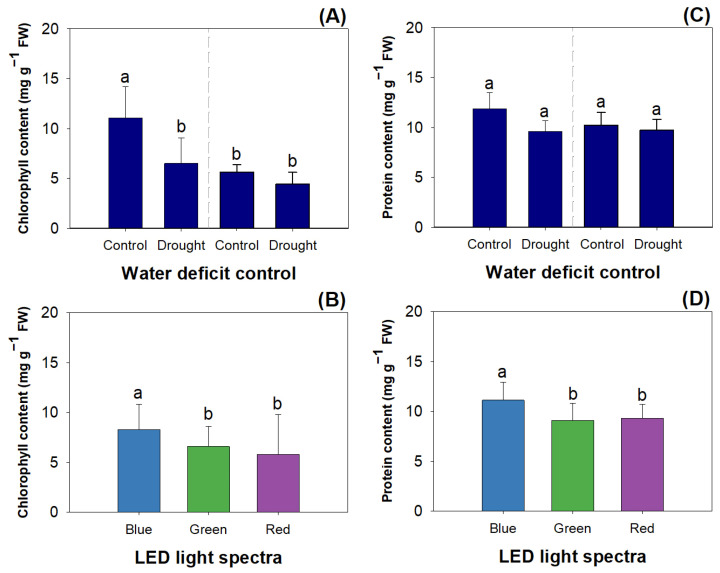
The unique effects of water deficit (**A**,**C**) and LED spectra (**B**,**D**) on chlorophyll content (**A**,**B**) and protein content (**B**,**D**) in shoots of *R. rubescens* seedlings. Different letters marked above error bars (standard errors) represent significant differences according to Tukey’s test at the 0.05 level.

Mowing and drought had an interactive effect on the chlorophyll content, while LED spectra had the main effect on it (Table 3). Controlled seedlings without a drought threat showed higher chlorophyll content than those suffering drought before mowing and post-mowing seedlings (Figure 3A). The blue light resulted in a higher chlorophyll content than the green and red lights (Figure 3B). 

Mowing, LED spectra, and drought had the main effects on the soluble protein content (Table 3). Mowing reduced the protein content by 16.48% from 10.73 mg g^−1^ to 8.97 mg g^−1^ (Figure 3C). Drought decreased the protein content by 21.41% (control, 11.03 ± 1.55 mg g^−1^; drought, 8.67 ± 1.32 mg g^−1^) (Figure 3C). Blue light resulted in a higher protein content (11.12 ± 1.79 mg g^−1^) compared to green (9.11 ± 1.71 mg g^−1^) and red lights (9.31 ± 1.43 mg g^−1^) (Figure 3D). 

Drought reduces the cumulative levels of chlorophyll and protein in stressed plants because a water deficit impairs nitrogen (N) assimilation and utilization [45]. Mowing has also been reported to decrease the chlorophyll content in *Lactuca sativa* seedlings, thereby reducing photosynthetic ability [46]. Exposure to blue light was also found to increase chlorophyll and protein contents in *Gerbera jamesonii* plantlets [47] and *Quercus variabilis* seedlings [48]. These can all be attributed to a widely recognized phenomenon that the blue-light spectrum can be well perceived and absorbed by plants as a signal to upregulate N uptake and utilization. 

**Figure 4:**
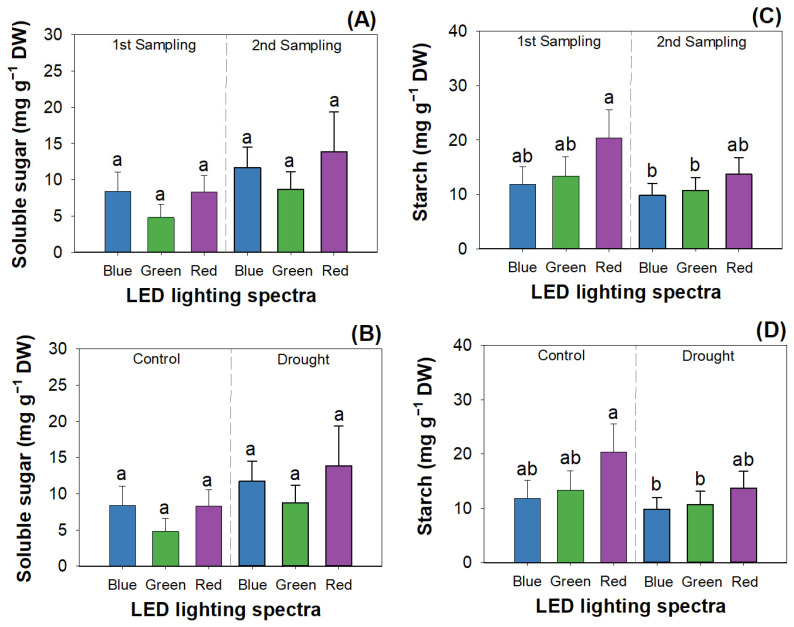
Combined effects of LED spectra and mowing (**A**,**C**) and LED spectra plus water deficit treatment (**B**,**D**) on soluble sugar content (**A**,**B**) and starch content (**C**,**D**) in shoots of *R. rubescens* seedlings. Different letters marked above error bars (standard errors) represent significant differences according to Tukey’s test at the 0.05 level.

The factors of mowing, LED spectra, and drought all exerted the main effects on soluble sugar content. Either mowing or drought stress had a combined effect with LED spectra on the starch content (Table 3). Mowing increased the soluble sugar content by 59.35% across mowing from 7.17 mg g^−1^ in the first sampling to 11.43 mg g^−1^ in the second (Figure 4A). The soluble sugar content under green light (6.76 ± 2.66 mg g^−1^) was lower than that under the blue (10.05 ± 2.95 mg g^−1^) and red lights (11.09 ± 4.63 mg g^−1^) (Figure 4B). Drought increased the soluble sugar content (control, 7.57 ± 2.57 mg g^−1^; drought, 11.03 ± 4.14 mg g^−1^).

Before mowing, seedlings showed a higher starch content in the red light than in green and blue lights (Figure 4C). Mown seedlings did not show any difference in the starch content among different LED spectra, but the starch contents in mown seedlings exposed to blue and green lights were both lower than the content in the first sampled seedlings exposed to red light. The starch content in the first sampled seedlings exposed to red light was higher than that in mown seedlings exposed to blue and green lights (Figure 4D). 

In this study, results suggest that drought resulted in a decline in starch content but, meanwhile, also increased sugar accumulation. Starch is a substance that is hydrolyzed to fuel resistance to water deficit, and soluble sugars are its primary gross product [49]. LED spectra can interact with drought and, together, affect the starch content. Compared to drought stress, well-watered conditions can increase starch content in the red light relative to that in blue and green lights. However, soluble sugars did not respond to this interaction. These results together suggest that red-light exposure can increase the synthesis and accumulation of starch compared to green or blue light when facing a drought event. We do not consider that red light exposure alleviated starch hydrolyzation because the sugar content was not changed by exposure to the LED spectrum. Red light was also reported to increase starch content in *Doritaenopsis* plants [50] but failed to promote starch accumulation in alfalfa [19]. These contrasting responses of starch content to red light exposure can be controlled by stomatal conductance, which determines starch metabolism via the synthesis of a counter ion to potassium [51]. Both the sugar and starch contents were improved by mowing, which suggests an elevation in carbohydrates and accords with common responses in meadow plants [52]. We do not consider that mowing can practically modify drought resistance by controlling carbohydrate metabolism. 

**Figure 5:**
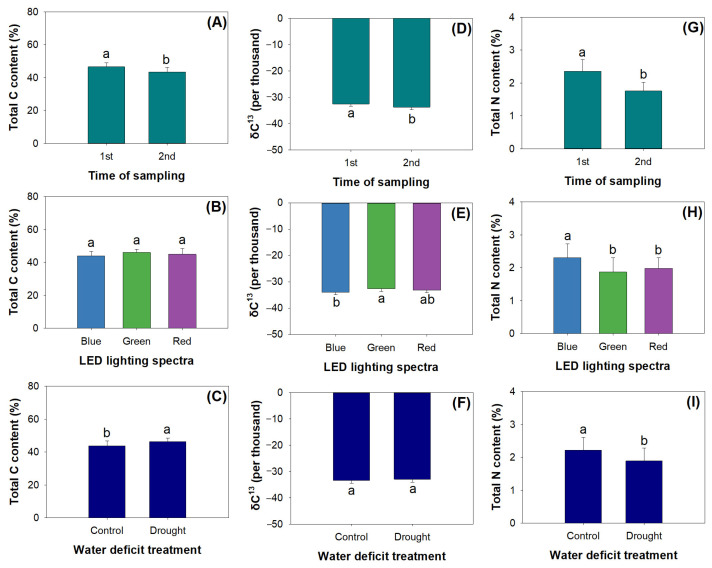
Unique effects of mowing (**A**,**D**,**G**), LED spectra (**B**,**E**,**H**), and water deficit treatment (**C**,**F**,**I**) on total carbon (**C**) content in shoots of *Rabdosia rubescens* seedlings. Different letters marked above errors bars (standard errors) present significant differences according to Tukey’s test at the 0.05 level.

The factors of mowing, LED spectra, and drought had the main effects on the total carbon © content, δ^13^C, and total N content (Table 3). Mown seedlings had a lower total C content compared to unmown ones (Figure 5A). Variations in the LED spectra did not cause any significant changes in the total C content (Figure 5B). Drought increased the total C content (Figure 5C). δ^13^C was lowered in post-mown seedlings compared to in those before mowing (Figure 5D). Compared to the δ^13^C in blue light, that in green light was 3.87% higher, and that in red light did not show significant differences (Figure 5E). However, drought did not change the δ^13^C. Again, mown seedlings had a lower total N content in shoots (Figure 5G). Blue light resulted in a higher total N content than green and red lights (Figure 5H). Drought decreased the total N content by 14.97% (Figure 5I). All these changes resulted from the responses of biomass and nutrient concentration to treatments because nutrient content is the product of biomass and concentration. 

Drought increased the total C content, which accorded with an increase in soluble sugars. This resulted from the decline in dry mass caused by water deficiency because the C frame was reduced while C assimilation did not change. This could also explain the decline in the total N content in drought. An increase in total C was also found in alfalfa [19]. However, δ^13^C in this study was not increased by drought as was reported for alfalfa and *Podocarpus macrophyllus* [4]. We surmise that *R. rubescens* had a strong sprouting ability, which overcame the limited effect of drought stress. Drought did not interact with mowing or LED spectra under C and N cycling. Blue light promoted N uptake and increased the total N content in shoots, which concurs with findings on *Aronia melanocarpa* [53] and *Acer truncatum* [54]. By contrast, blue light stimulated gas exchange and lowered water-use efficiency (WUE) due to the decrease in δ^13^C. This was in accordance with findings collected from studies on alfalfa [19], both demonstrating that blue light stimulated gas conductance and lowered water-use efficiency [55]. The declines in the total C content, total N content, and δ^13^C together demonstrate that mowing cannot be suggested for the culture of *R. rubescens* because mown plants show weakened C-N acquisition and WUE. 

### 2.4. Antioxidant Activity

**Figure 6:**
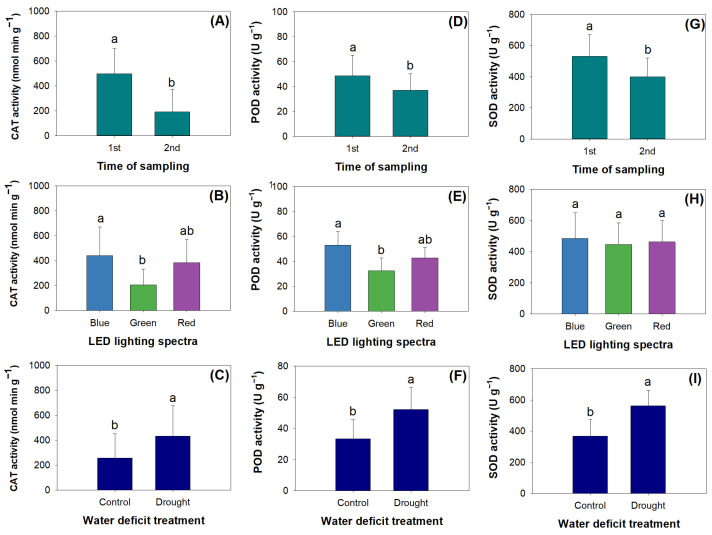
Unique effects of mowing (**A**,**D**,**G**), LED spectra (**B**,**E**,**H**), and water deficit treatment (**C**,**F**,**I**) on enzymatic activities of catalase (CAT) (**A**–**C**), peroxidase (POD) (**D**–**F**), and superoxide dismutase (SOD) (**G**–**I**) in shoots of *R. rubescens* seedlings. Different letters marked above error bars (standard errors) represent significant differences according to Tukey’s test at the 0.05 level.

The factors of mowing, LED spectra, and drought had the main effects on catalase activity (CAT), peroxidase activity (POD), and superoxide dismutase activity (SOD) (Table 4). Mowing decreased the activity of three enzymes (Figure 6A,D,G). Blue light resulted in higher CAT (Figure 6B) and POD (Figure 6E) compared to green light, but this difference was not significant for SOD (Figure 6H). Drought increased CAT, POD, and SOD (Figure 6C,F,I). 

### 2.5. Regression of Medicinal Quality against Plant Parameters

Regressions using three models together indicated that δ^13^C (models 1–3) and CAT (models 2–3) were two plant parameters that contributed to the regression of oridonin content (Table 5). In model 1, stepwise regression indicated δ^13^C is a unique parameter that makes a negative contribution. In model 2, forward linear regression indicated both δ^13^C and CAT have negative contributions, and the estimate of δ^13^C contribution was lower by 0.07 compared to that of CAT. In model 3, the backward regression model also indicated two negative contributions from δ^13^C and CAT, and again, the estimate of δ^13^C was lower. 

These findings suggest that fluent gas exchange from high stomatal conductance (low δ^13^C) together with a lowered stressed state (low CAT) are two obligatory preconditions to producing high quantities of oridonin. Stomatal conductance made a lower contribution to fat formation compared to CAT, indicating that good acclimation to drought mattered for oridonin production; however, gas exchange generated a stronger driving force marked by δ^13^C. 

## 3. Materials and Methods

### 3.1. Plant Materials

*Rabdosia rubescens* seeds were sterilized by spraying 5% (*w*/*w*) potassium permanganate before soaking in distilled water for 12 h. Seeds were incubated on moist sands at 23 °C in a plant-growing chamber (Binglin Electronic S&T Inc., Shanghai, China). Germinated plantlets were transplanted to growing media (perlite and peat, 1:3, *v*/*v*) that were filled to the cavities (diameter and height, 7 cm × 13 cm) and embedded in a growing tray. A total of 32 plantlets were transplanted into the cavities of a planting tray. Controlled-release fertilizer (CRF) granules (N-P_2_O_5_-K_2_O, 14-13-13; Osmocote, the Scotts Co., Marysville, OH, USA) were mixed with growing media at a rate of 0.54 g N pot^−1^ [56]. Plantlets were fully watered and placed in tanks (40 cm width × 60 cm length) to be raised by sub-irrigation [17,18]. A total of 72 tanked planting trays (2304 plantlets) were prepared in this study. Seedlings were cultured under conditions with a temperature ranging from 17 to 34 °C (night/day) and relative humidity of 51.71 ± 14.20% (ranging from 33 to 84%). The whole experiment was conducted over 120 d since the transplant. 

### 3.2. LED Light Illumination

One week after transplant, the seedlings were planted in plastic pots (11.5 cm top diameter × 9.5 cm height × 7.5 cm bottom diameter) whose sizes were employed as those used by Gao et al. [48]. The seedlings used for transplant were required to be uniform in size with a height of ~4 cm. Four individual seedlings per tray were planted in one pot, and all 32 seedlings per tray were planted in eight pots, which were placed in a tank exposed to LED panels. Mixed-wavelength spectra were employed for seedling illumination [48]. Three types of LED spectra were provided as visible colors: red, blue, and green [17]. Wavelength components were adopted from the regimes of Wang et al. [57]. The red-light LED spectrum comprised red-, green-, and blue-light wavelengths at 71.7%, 13.7%, and 14.6%, respectively; the green-light LED spectrum comprised 26.2%, 56.4%, and 17.4% red-, green-, and blue-light wavelengths, respectively; the blue-light LED spectrum comprised wavelengths of 17.8% red light, 33.7% green light, and 48.5% blue light. All the LED panels were equipped to a height of about 50 cm for overhead trays. The photosynthetic photon flux density (PPFD) ~40 cm beneath the surface of the panel was determined to be about 63.8 ± 2.27 µmol m^−2^ s^−1^. The PPFD at the understory layer could fall in the range between 3.60 and 175.67 µmol m^−2^ s^−1^ [16]. 

### 3.3. Drought Treatment Implementation

At 14 d after transplant, seedlings were thinned, leaving only one in a pot to make sure that the left seedlings were healthy and strong. All the left seedlings received drought treatment immediately post-thinning. Half of the seedlings were watered with a frequent supplement, and the tanked water was replenished every two days to make sure the root rhizosphere was moist all the time. These seedlings were marked as the control, and they were well watered through a sub-irrigation system with water absorption with pore movement upwards. The other half of the seedlings were treated by simulated drought treatment. Every pot was sealed by package tapes on the top to prevent transpiration from the surface of the growing media. During drought treatment, all the seedlings continuously received LED lighting to maintain their basic growth. All the LED lighting spectra were provided for all the seedlings during their exposure to drought. 

According to practical experience, *R. rubescens* seedlings can be raised during the growing season for three months in rangeland [30]. Therefore, the water deficit condition was simulated for the delivery of water to a volume of 470 mL in 90 d. This was determined according to an estimate made by He et al. [4] using drought-event data from 191 meteorological stations across nine provinces of China over a time of 113 years [58]. Drought treatment was implemented up to 105 d after transplant. 

### 3.4. Mowing Implementation and Sampling

Seedlings were mown 46 d after transplant when most sprouts had grown to touch the surface of the LED panels. This length of time was also in accordance with that for the culture of alfalfa [19]. All the sprouted shoot parts were removed, leaving the intact parts of perennial roots. Seedlings were first sampled 39 d after transplant when they had not yet been mown. Two technical replicates were employed using two tanks for sampling to eliminate the possible effect of a decline in density on the left seedlings. Four seedlings were randomly chosen from one tank, with eight in total from two tanks. Only sprouted shoots were sampled. Mown seedlings were sampled for the second time 120 d after transplant. 

### 3.5. Experimental Design and Arrangement

**Figure 7:**
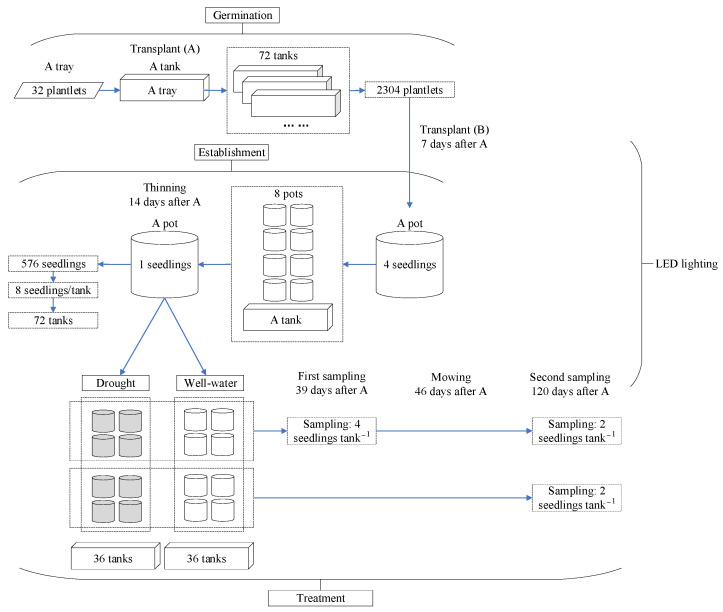
The overall arrangement of the experimental layout.

The overall arrangement of the experimental layout is shown in Figure 7. A total of 72 tanks of potted seedlings were used as study materials for a combined factorial design for LED spectra (degrees of freedom (*df*) of 2), drought (*df* = 1), and mowing (*df* = 1). Therefore, a total of 12 combined treatments (3 LED × 2 droughts × 2 mown) were assigned to 72 tanked pots, six tanks were assigned as replicates, and eight potted seedlings were assigned as repeatedly measured units per tank. For a combined treatment, every two tanks were assigned as a basic sampling unit whose measures were averaged for a technical repeat, and three repeats were assigned for three replicates for combined treatment. At each sampling, all eight seedlings were measured for their height and RCD. Subsequently, four of them were randomly chosen and used to determine physiological parameters as fresh samples. The other four of them were bulked and used to measure dry mass, which was determined using dried samples.

### 3.6. Parameter Determination

Potted seedlings were weighed from the 10th day after the commencement of drought treatment. Seedlings were grouped by combined treatments across three LED spectra and two water conditions, leaving 12 tanked pots for each treatment. Eight tanks of potted seedlings were further chosen by random selection and labeled for pot weighing. Every pot was measured for the weight of potted substrates together with the plant. Eight pots per tray were weighted, and their mean was used as the average weight for the tray. As a result, eight trays of potted seedlings were fixed as eight replicated units which were continuously weighed on the following days after transplant, i.e., 25 d, 32 d, 39 d, 53 d, 63 d, 70 d, 77 d, 84 d, 91 d, 98 d, 105 d, and 112 d. Because half of the seedlings were obligated to be mown at 46 d after transplant, four tanks of eight labeled replicates from a combined treatment were subjected to mowing, and the other four remained unmown. 

The plant diameters were determined using fresh samples, including chlorophyll and the soluble protein contents of CAT, POD, and SOD. The chlorophyll and protein contents were determined using a method adapted from Wei et al. [23]. A 0.05 g sample was mixed with 2.5 mL of dimethyl sulfoxide and incubated over boiling water. Chlorophyll-a and -b were determined using a spectrophotometer at 663 nm and 645 nm, respectively. In this study, the chlorophyll content was determined as the total weight of the chlorophyll-a and -b contents. Another sample at a weight of 0.1 g was ground and dissolved in phosphate buffer (pH 7.5). The solution was centrifuged at 3000 rpm for 10 min and treated using 0.1 mL of Folin reagent, and the soluble protein was measured at 650 nm. CAT, POD, and SOD were assayed using a microplate reader (Multiskan GO, ThermoFisher Sci., Shanghai, China) [59]. The specific details of the determination of CAT, POD, and SOD can be found in Sima et al. [60], Wang et al. [61], and Sima et al. [60], respectively. 

Another four samples per tray were dried in an oven at 70 °C for 72 h; then, their dry mass was weighed. Dried samples were used to determine the oridonin content, soluble sugar and starch contents, total C content, δ^13^C, and total N content. Oridonin was determined using a method adapted from Harris et al. [34]. The soluble sugar and starch contents were determined using a spectrophotometer (UV-Visible 8453 analyzer, Agilent Inc., SF, CA, USA) [49]. The total C content was determined using an element analyzer (EA-3000, Boaying Tech., Shanghai, China). C-isotope discrimination was performed using samples that were smashed and passed through a 1 mm sieve. δ^13^C was determined using Finnigan Delta^plus^ XP (ThermoFisher Scientific Branch, Shanghai, China) following an equation [19]:

(1) $$\delta^{13}C\left(‰\right)=\left(\frac{R_{Sample}}{R_{Standard}}-1\right)\times 1000$$

where *R_Sample_* and *R_Standard_* are the ratios of proportional ^13^C to ^12^C in samples and the Pee Dee Belemnite standard, respectively. The total N content was determined using the Kjeldahl method [62]. 

### 3.7. Statistical Analysis

The data were tested, and the normal distribution pattern and homogeneity of variance were confirmed. The data were analyzed for a split–block design using mixed-model analysis of variance (ANOVA) with three statistical replicates (two technically repeated trays per combined treatment). That is, the drought treatment was assigned as the main block (*df* = 1, drought vs. control), with LED spectra as sub-blocks (*df* = 2, red-, green-, and blue-colored spectra). Mowing caused two samplings to be arranged as two repeated measures (*df* = 1, first and second samplings). Each statistical repeat was assigned to two trays as a basic unit of sampling, and each tray was assigned as a technical repeat. When a significant interaction was indicated by ANOVA, the results were compared using Tukey’s test, and critical significance was determined at the *α* = 0.05 level. Oridonin content was taken as the goal parameter that could reflect the quality of forage. Oridonin content was regressed in multiple linear regression models against all plant parameters. Three models were used, namely stepwise, forward, and backward regressions, with an aim to eliminate the bias of over-unique model regression. Only parameters that were estimated to make significant contributions to the regression across all three models were screened as the chosen parameters to predict oridonin content. 

## 4. Conclusions

*Rabdosia rubescens* seedlings were cultured under combined treatments of mowing and LED exposure under a water deficit condition. In terms of the negative effects of drought on growth and oridonin content, mowing did not show the expected impact in helping plants to cope with the consequences of a water deficit, and neither did its interaction with LED lighting. Mowing’s main effect was to increase medicinal quality by enhancing oridonin content, but it also reduced C and N accumulation and water-use efficiency. LED lighting also failed to enhance the ability to cope with drought according to seedling parameters. The red-light spectrum was better than blue or green light in benefiting starch accumulation only under well-watered conditions. Green light can improve water-use efficiency but impair the nitrogen and oridonin contents compared to blue light. Overall, neither mowing nor LED lighting can be effectively used as an instrument to help cope with drought in the culture of *R. rubescens*. If a higher oridonin content is desired, either mowing or the blue-light spectrum could be chosen as a promoter. For predicting the medicinal quality of *R. rubescens*, lower levels of both δ^13^C and CAT can be taken as predictors, and a couple of indicators can be used to predict a high content of oridonin. 

## Figures and Tables

**Table 1:** *p* values from analysis of variance of combined effects of mowing, LED spectra, and drought on growth, biomass production, and quality in *Rabdosia rubescens* seedlings.

Source of Variation	Seedling Growth, Dry Mass Production (DM), and Quality			
	Height	RCD ^1^	Oridonin	DM
Mowing (M)	**<0.0001 ^2^**	0.1489	**0.0036**	0.2246
LED (L)	**0.0428**	0.0625	**0.0409**	**0.0052**
Drought (D)	**0.0007**	**0.0081**	**0.0013**	**0.0018**
M × L	0.5187	0.3628	0.9790	**0.0494**
M × D	0.1142	0.1223	0.7481	0.3913
L × D	0.4407	0.3431	0.1193	0.2193
M × L × D	0.6022	0.3964	0.9688	0.7512

^1^ RCD, root–collar diameter; ^2^ values in bold font indicate significant effects.

**Table 2:** A summary of studies quantifying oridonin content in different organs from *Rabdosia rubescens* and *Isodon enaderianus* at varied times of year in fields of different habitats in China.

Source	Species	Provincial Habitat	Sampling Time	Organ	Oridonin Content (% DW)
Guo et al. [41]	*Rabdosia rubescens*	Henan	Unknown	Whole plant	0.550
		Guangxi			0.540
		Jiangxi			0.590
		Sichuan			0.580
Cui et al. [42]	*R. rubescens*	Henan	July	Whole plant	0.470
			August		0.620
			September		0.630
			October		0.450
Yang et al. [43]	*R. rubescens*	Taiwan	Unknown	Whole plant	0.468
Harris et al. [34]	*Isodon enaderianus QD*	Guizhou	October and November	Leaves	0.000
	*I. enanderianus* TR	Guizhou			0.000
	*I. henryi* XN	Henan			0.000
	*I. japonicus* XX	Henan			0.752
	*I. lophanthoides* var. *micranthus* TR	Guizhou			0.000
	*I. rubescens* HB	Henan			0.394
	*I. rubescens* JY	Henan			0.317
	*I. rubescens* QY	Henan			0.256
	*I. rubescens* XX	Henan			0.272
	*I. rubescens* YC	Hubei			0.150
	*I. enaderianus QD*	Guizhou	August		0.000
	*I. enanderianus* TR	Guizhou			0.000
	*I. henryi* XN	Henan			0.000
	*I. japonicus* XX	Henan			0.211
	*I. rubescens* HB	Henan			0.200
	*I. rubescens* JY	Henan			0.289
	*I. rubescens* QY	Henan			0.263
	*I. rubescens* XX	Henan			0.174
	*I. rubescens* YC	Hubei			0.160
Jian et al. [44]	*I. rubescens* (HEMSLEY) H. Hara	Henan	Unknown	Root	0.008

**Table 3:** *p* values from the analysis of variance for the combined effects of mowing, LED spectra, and drought on physiological parameters in *R. rubescens* seedlings.

Source of Variation	Physiological Parameters						
	Chlorophyll	Protein	Sugar	Starch	TC ^1^	δ^13^C	TN ^2^
Mowing (M)	**<0.0001 ^3^**	**<0.0001**	**<0.0001**	**<0.0001**	**0.0026**	**0.0005**	**<0.0001**
LED (L)	**0.0262**	**0.0002**	**0.0019**	**<0.0001**	0.1865	**0.0066**	**0.0050**
Drought (D)	**0.0005**	**<0.0001**	**0.0009**	**<0.0001**	**0.0110**	0.1078	**0.0031**
M × L	0.6250	0.0784	0.5941	**0.0292**	0.7660	0.2914	0.8615
M × D	**0.0260**	0.7045	0.0886	0.0795	0.6220	0.1614	0.8613
L × D	0.1386	0.4164	0.1599	**0.0329**	0.5425	0.1264	0.5941
M × L × D	0.2129	0.6399	0.3737	0.5574	0.7867	0.7391	0.9806

^1^ TC, total carbon content; ^2^ TN, total nitrogen content; ^3^ values in bold font indicate significant effects.

**Table 4:** *p* values from analysis of variance for the combined effects of mowing, LED spectra, and drought on antioxidant activity in *R. rubescens* seedlings.

Source of Variation	Antioxidant Activity		
	CAT ^1^	POD ^2^	SOD ^3^
Mowing (M)	<0.0001	0.0047	0.0044
LED (L)	0.0180	0.0007	0.7400
Drought (D)	0.0126	<0.0001	<0.0001
M × L	0.4685	0.0852	0.8261
M × D	0.7877	0.6173	0.9715
L × D	0.8174	0.2702	0.7273
M × L × D	0.7202	0.6666	0.2622

^1^ CAT, catalase activity; ^2^ POD, peroxidase activity; ^3^ SOD, superoxide dismutase activity.

**Table 5:** Parameter estimate in multiple linear regression models for oridonin content against plant parameters in *R. rubescens* seedlings.

Variable	Model 1 ^1^			Model 2 ^2^			Model 3 ^3^		
	Parameter	SE ^4^	*p*	Parameter	SE	*p*	Parameter	SE	*p*
Intercept	−1.9291	1.2884	0.1436	0.2539	1.4838	0.8653	−1.4182	1.2337	0.2589
C13 ^5^	−0.1125	0.0388	0.0066	−0.0757	0.0364	0.0464	−0.0911	0.0368	0.0187
CAT ^6^				−0.0004	0.0002	0.0496	−0.0004	0.0002	0.0176

^1^ Model 1, stepwise regression model; ^2^ Model 2, forward linear regression model; ^3^ Model 3, backward linear regression model; ^4^ SE, standard error; ^5^ C13 delta C-13 discrimination; ^6^ CAT, catalase activity.

## Data Availability

Not applicable.

## Supplementary Materials

The following supporting information can be downloaded at: https://www.mdpi.com/article/10.3390/plants12183189/s1. Figure S1: Dynamics of weight in potted *Rabdosia rubescens* seedlings exposed to combined LED spectra and drought stress. Each column presents an average of eight replicates (tanked pots of seedlings), and error bars show standard errors. Different letters indicate significant differences according to Tukey’s test at the 0.05 level on a sampling day. Red, Blue, and Green denote the LED light colors; Ctrl, a well-watered control; Drought, drought stress. Days mark post-transplant periods.
