# Supplementary material for: Light-Emitting Diodes Modify Medicinal Quality of Mown Rabdosia rubescens, with Changes in Growth, Physiology, and Antioxidant Activity, under Drought Stress
Source: Plants (Basel). 2023 Sep 6;12(18):3189. doi: 10.3390/plants12183189 (PMC10536318; doi:10.3390/plants12183189)
Supplement: Supplementary file 1 [file plants-12-03189-s001.zip › plants-2545366-supplementary.pdf]

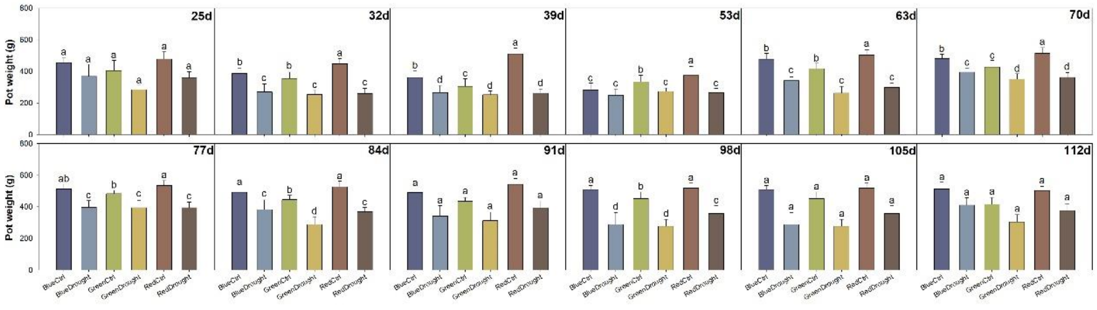


**Figure S1.** Dynamics of weight in potted *Rabdosia rubescens* seedlings exposed to combined LED spectra and drought stress. A column presents an average of eight replicates (tanked pots of seedlings) and error bars mark standard errors. Different letters indicate significant difference according to Tukey test at 0.05 level in a sampling day. Red, Blue, and Green are abbreviations of LED light colors; Ctrl, a well-watered control; Drought, drought stress. Days mark post-transplant periods.
